# Severe Kawasaki Disease and Thrombocytopenia: A Case Report

**DOI:** 10.7759/cureus.42916

**Published:** 2023-08-03

**Authors:** Ghizlane Souni, Ghanam Ayad, Aziza Elouali, Maria Rkain, Abdeladim Babakhouya

**Affiliations:** 1 Pediatrics, Mohammed VI University Hospital, Oujda, MAR; 2 Pediatrics, Faculty of Medicine and Pharmacy of Oujda, Mohammed First University, Oujda, MAR

**Keywords:** thrombocytosis, hematologic abnormality, coronary artery abnormalities, thrombocytopenia, kawasaki disease

## Abstract

Kawasaki disease (KD) or lymphocutaneous mucosal syndrome is a medium vessel vasculitis of unknown mechanism, which mainly affects the coronary arteries. The diagnosis is mainly based on clinical criteria. Biologically, thrombocytosis is the usual biological disturbance of this disease. Herein, we report a 2-year and 10-month-old girl, who was admitted to our department for a febrile rash that had been evolving for seven days prior to her admission. Clinical examination revealed a rash involving the entire body, conjunctivitis, cheilitis, and a strawberry tongue. A biological inflammatory syndrome could be identified with thrombocytopenia at 91,000/mm^3^. The patient received intravenous immunoglobulins and acetylsalicylic acid with a favorable evolution and complete resolution of thrombocytopenia.

## Introduction

Kawasaki disease (KD) is an acute vasculitis of unknown etiology that affects medium-sized blood vessels with a particular predilection for coronary arteries. It is the leading cause of acquired heart disease in children in industrialized countries [1]. The distribution of KD worldwide is not homogeneous, with a preference for children of Japanese origin, with an incidence rate of 264.8/100,000 children under five years old [2]. The diagnosis is based solely on clinical criteria. Biologically, KD is characterized by a marked elevation of inflammatory markers. Thrombocytosis is characteristic of Kawasaki disease and is generally observed during the second or third week of the disease. Thrombocytopenia is therefore an unusual situation and has rarely been reported [3]. Here, we report a case of a child with Kawasaki disease, initially presenting with thrombocytopenia.

## Case presentation

We report a 2-year and 10-month-old girl with no notable medical history, who was admitted to our department for a febrile rash that had been evolving for seven days prior.

Her admission laboratory results revealed leukocytosis at 21,770/mm^3^ (with neutrophils at 17,220 element/mm^3^; lymphocytes at 3,890 element/mm^3^; and monocytes at 990 element/mm^3^), hypochromic microcytic inflammatory anemia (hemoglobin was at 10.4 g/dl; mean corpuscular volume (MCV) at 78.60 fl; mean corpuscular hemoglobin concentration (MCHC) at 27.50 g/dl; and ferritin level at 443 mg/l), thrombocytopenia at 91,000/mm^3^, elevated C-reactive protein (CRP) at 403 mg/l, accelerated sedimentation rate at 99 mm, and significantly elevated brain natriuretic peptide (BNP) at 2641 IU. ECG was normal and troponin levels were normal. Serologies were also performed for cytomegalovirus (CMV), Epstein-Barr virus (EBV), and TORCH (toxoplasmosis, rubella cytomegalovirus, herpes simplex, and HIV) infections, and were negative. The prothrombin time was prolonged, and the peripheral blood smear revealed the presence of schistocytes, helmet cells, and platelet clumps.

On the paraclinical level, the cardiac ultrasound revealed coronary dilation. Based on the clinical criteria, we diagnosed her with Kawasaki disease after discussing potential causes of thrombocytopenia, including probable disseminated intravascular coagulation and macrophage activation syndrome.

In terms of treatment, the patient received an emergency infusion of intravenous immunoglobulin (IV Ig) at a dose of 2 g/kg, along with anti-inflammatory acetylsalicylic acid (80 mg/kg/day).

**Figure 1:**
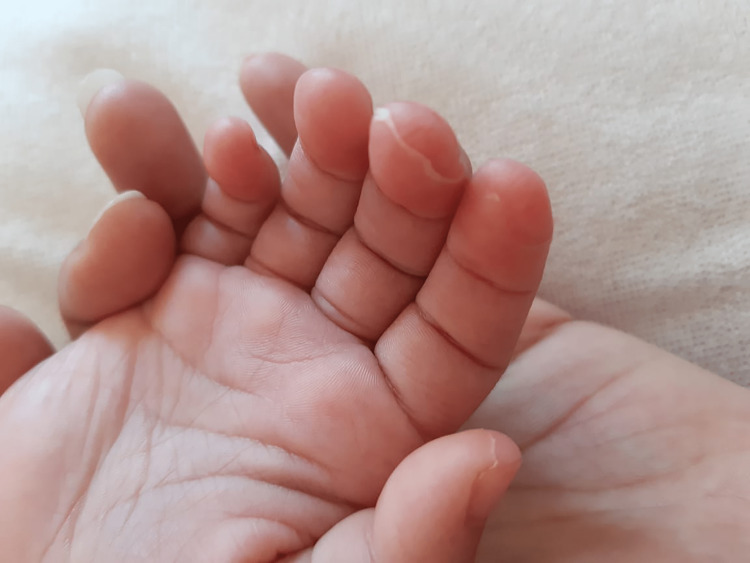
Glove-like desquamation, typical of Kawasaki disease, has been observed in our patient

Two days later, due to persistent fever, our patient received a second dose of IV Ig and was reassessed the following day. Clinically, she achieved afebrile status from the first day, and her platelet count increased (from an initial 91,000 to 142,000) while CRP levels showed a decreasing trend (from an initial 403 to 133). Three days later, desquamation appeared on the palms of her hands and the soles of her feet, in a glove-like fashion, which is typical of Kawasaki disease (Figure 1).

**Figure 2:**
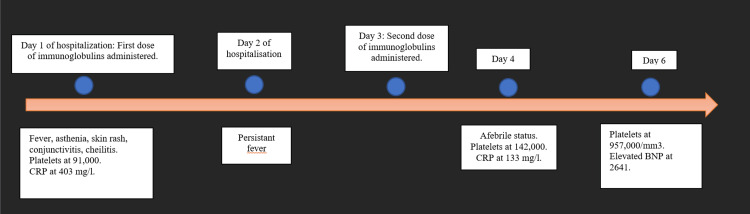
Chronological clinical and laboratory evolution of our patient CRP: C-reactive protein; BNP: brain natriuretic peptide

Her platelet count reached 957,000/mm^3^, and CRP levels were at 8.31 mg/l. Figure 2 shows the chronology of different events related to the management of our patient.

## Discussion

Kawasaki disease (KD) is an acute vasculitis whose cause remains unknown. Various theories have been proposed based on epidemiological, pathological, and demographic data. Epidemiological data suggest an infectious cause for KD, given its resemblance to other pediatric infectious diseases, its seasonality, and its often-epidemic presentation. Various pathogens have been implicated and subsequently ruled out, as no single infectious agent could be identified as the causal factor. There is evidence associating seasonality with atmospheric currents, supporting the hypothesis that an airborne agent could trigger an immune response, leading to KD in genetically predisposed children. Different genes associated with increased susceptibility to the disease, or a more aggressive course, have been identified. These include genes encoding regulatory molecules of the immune response, vascular growth factors, the immunoglobulin G receptor, or human leukocyte antigens (HLA) [2].

The diagnosis of KD is primarily based on well-defined clinical criteria. These criteria include the presence of persistent fever for at least five days and the existence of at least four of the following five clinical signs: extremity swelling, rash, conjunctival hyperemia, cheilitis, and cervical lymphadenopathy, typically unilateral, with a size greater than or equal to 1.5 cm in diameter [3]. These criteria are partly subjective, and currently, there is no specific biological marker available. However, certain clinical signs, although not included in the diagnostic criteria, should raise suspicion of KD. These include behavioral changes with significant irritability and the presence of erythema at the site of a Bacillus Calmette-Guérin (BCG) scar [4].

There are no specific biological markers for this disease, but it is generally associated with a marked elevation of inflammatory markers, including thrombocytosis, which is considered a minor criterion for the diagnosis of the disease according to the Japanese guidelines [3]. Thrombocytosis is typically observed during the second or third week of the disease. On the other hand, the association with thrombocytopenia is an unusual form of KD and has been rarely reported in the literature, with a prevalence of 3% in a Chinese series [5]. The complications and mortality associated with this disease are mainly due to cardiac involvement, which manifests as coronary artery dilatation [4].

The current recognized treatment as the gold standard is the use of intravenous immunoglobulins (IVIG) at a dose of 2 g/kg, along with an anti-inflammatory dose of acetylsalicylic acid (80-100 mg/kg/day) in the acute phase. Subsequently, most North American teams reduce the dose of acetylsalicylic acid to an antiplatelet dose (3-5 mg/kg/day) after 48 to 72 hours of defervescence and continue it for a duration of six weeks or longer in the presence of coronary abnormalities [6]. The characteristic evolution is marked by a typical desquamation of the extremities in a glove-like pattern.

Several studies have investigated the association between KD and thrombocytopenia. A prospective study conducted in France, involving 123 pediatric departments over one year, found that among 142 children with KD, 44 initially presented with thrombocytopenia or developed it during treatment without clinical manifestations before the appearance of thrombocytosis, which usually occurs around the third week of the disease [7]. Nafech et al. examined 31 patients with KD and thrombocytopenia published in the English literature and found that this combination was more common in girls and young children. The authors further noted that thrombocytopenia appeared in the acute phase of the disease, typically on days 5 to 12. Among the 31 patients with KD and thrombocytopenia, 14 developed coronary aneurysms (45%) while myocardial infarction and death were observed in four and two cases, respectively. Niwa et al. reported 303 patients with KD, among whom 10 patients had concurrent thrombocytopenia. They found that six out of 10 patients with thrombocytopenia (60%) developed aneurysms while among the remaining 293 patients, only 8.9% developed aneurysms. Thus, thrombocytopenia may be a risk factor for coronary artery aneurysms (CAA) during the acute phase of KD [3]. Indeed, our patient presented with thrombocytopenia without hemorrhagic signs on the fourth day of the disease, which was followed, after treatment, by significant thrombocytosis and complicated by a coronary aneurysm. There are various theories regarding the mechanism of thrombocytopenia in individuals with the disease. It has been hypothesized that thrombocytopenia is secondary to platelet consumption induced by coagulation, as evidenced by abnormal coagulation tests and the presence of disseminated intravascular coagulation (DIC) in these patients while others attribute it to increased platelet destruction by immunoglobulin or non-immune mechanisms (i.e., binding of an infectious agent to platelets) [3]. As no coagulation studies were performed in our patient, the possibility of an underlying low-grade consumption process as a cause of thrombocytopenia was identified.

## Conclusions

In conclusion, the clinician must be aware that thrombocytopenia can be a feature of Kawasaki disease in some cases, and it may indicate a more active condition than thrombocytosis. Children with KD and thrombocytopenia may have a higher risk of developing coronary artery aneurysms and myocardial infarction.
